# Chemotherapy-induced fulminant acute pancreatitis in pancreatic carcinoma: A case report

**DOI:** 10.3892/ol.2014.2316

**Published:** 2014-07-04

**Authors:** LINGHUI LU, YANNI LOU, HUANGYING TAN

**Affiliations:** Department of Traditional Chinese Medicine Oncology, China-Japan Friendship Hospital, Beijing 100029, P.R. China

**Keywords:** chemotherapy, fulminant acute pancreatitis, gemcitabine, capcitabine

## Abstract

Cases of chemotherapy-induced pancreatitis are rarely reported and among those diagnosed, the majority are mild and self-limiting. However, no previous cases of fulminant acute pancreatitis (FAP) induced by chemotherapeutic agents have been reported. The current study presents a case of FAP in a 62-year-old female on gemcitabine and capecitabine therapy. The patient was admitted to the China-Japan Friendship Hospital (Beijing, China) with the symptoms of acute pancreatitis two days after the completion of the first cycle of chemotherapy. Shock, hypoxemia and acute renal failure supervened, which resulted in mortality. As the common etiologies of pancreatitis were eliminated, a correlation between the incidence of FAP, and pancreatic cancer or chemotherapy, or both was suspected. Clinicians should be aware of this potential adverse effect when prescribing chemotherapeutic agents, particularly in patients with pre-existing risk factors for pancreatitis.

## Introduction

Fulminant acute pancreatitis (FAP) is a subgroup of severe AP, with rapid progression to multiorgan failure within 72 h and a high level of mortality. The common causes of pancreatitis include cholelithiasis, alcoholism, hyperlipidemia, overeating and idiopathic factors. In addition, pancreatic carcinoma and related chemotherapeutic agents are rare, but potentially serious causes, with low reported incidence rates of 0.4 (1) and 0.1–2% (2), respectively. The chemotherapeutic agents reported to induce pancreatitis include capecitabine, paclitaxel, bortezomib, vinorelbine and ifosfamide (3–14). The majority of these agents, however, are mild and self-limiting. The current study presents a case of a 62-year-old female with pancreatic carcinoma who developed FAP following the initial course of gemcitabine and capecitabine therapy. Patient provided written informed consent.

## Case report

**Figure 1:**
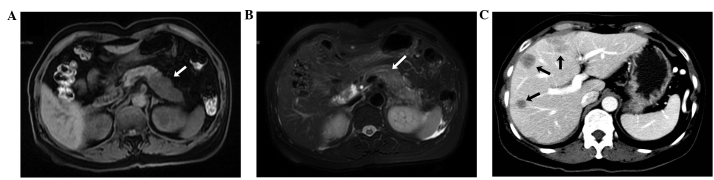
Magnetic resonance cholangiopancreatography imaging findings and diagnosis for (A) tumor in body and tail of pancreas and (B) interruption of the pancreatic duct, and (C) computed tomography imaging findings of the liver metastasis.

A 62-year-old female presented to the China-Japan Friendship Hospital (Beijing, China) with abdominal distension that had persisted for two months. Laboratory tests demonstrated an abnormal elevation of tumor markers, in particular that of carbohydrate antigen 19–9 (>1,000 U/ml). Abdominal computed tomography revealed a low density mass located in the body and tail of the pancreas, as well as multiple round hypodense regions in the liver. Pancreatic cancer was suspected, and therefore, a magnetic resonance cholangiopancreatography (MRCP) was performed. The results indicated that the pancreatic body and tail were distended and non-homogeneous, with abnormal signal intensity, abrupt interruption of the pancreatic duct and multiple nodular abnormal signals in the liver (Fig 1). A diagnosis of pancreatic carcinoma with liver metastasis was determined without pathological confirmation.

**Figure 2:**
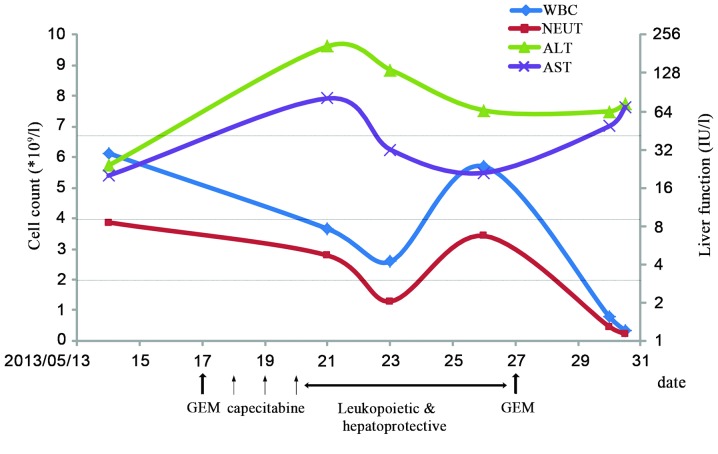
Chemotherapy progress. The green and purple lines represent the upper limit of normal ALT and AST levels (40 IU/l), respectively, with respect to the secondary axis denoting liver function. The blue and red lines represent the lower limit of normal WBC (4×10^9^/l) and NEUT (2×10^9^/l) levels, with respect to the primary axis. GEM, gemcitabine, WBC, white blood cell; NEUT, neutrophil, ALT, alanine transaminase; AST, aspartate transaminase.

On admission, the blood cell count and liver, kidney and pancreas function tests were normal. On May 15th, 2013, the patient, with a body surface area of 1.50 m^2^, received a chemotherapy treatment regimen consisting of 1.2 g gemcitabine intravenously on days one and eight, and 1,000 mg capecitabine orally twice a day, on days two to 15, of a 21 day cycle. However, during the course of the treatment, due to an elevated level of alanine transaminase (ALT) and a low white blood cell and neutrophil cell count, capecitabine was discontinued on the fifth day. Reduced glutathione and Leucogen were prescribed throughout the duration of the treatment. The subsequent gemcitabine treatment was delayed until the 11th day when the laboratory results had returned to normal, with the exception of the ALT level (65 IU/l; normal range, 0–40 IU/l). Following this cycle, the patient was discharged in a good condition. Two days later, on May 30th, the patient developed unexplained abdominal pain, which was followed by vomiting undigested food and clear gastric contents. Accompanied by profuse sweating, the pain worsened, particularly in the epigastric region, after a further two hours. The patient vomited once more, with ~2,000 ml of watery, hemorrhagic substances. Gradually, the patient became limp, weak and confused and was subsequently visited the Emergency Department of the China-Japan Friendship Hospital. The patient’s vital signs were measured and observed to be abnormal, with a body temperature of 38.3°C, a pulse rate of 145/min, a respiratory rate of 40/min, blood pressure of 53/30 mmHg and oxygen saturation at 60%. A normal level of consciousness was observed and heart and lung examinations were normal. The abdomen was flat with marked tenderness of the mid-upper section, where muscular spasms and rebound tenderness were observed. The laboratory tests revealed the following serum levels (normal ranges provided in parentheses): Amylase, 346 IU/l (28–100 IU/l); lipase, 936 IU/l (0–160 IU/l); and creatinine, 140.5 μmol/l (35–106 μmol/l). The blood cell count and liver function test results are shown in Fig. 2. Hypovolemic shock, myelosuppression and liver and kidney dysfunction were diagnosed, in addition to suspected AP. With continuous electrocardiography monitoring, the use of oxygen masks and protective isolation, the patient was admitted to the Department of Traditional Chinese Medicine Oncology and administered intravenous fluids, dopamine to maintain blood pressure, proton-pump inhibitors, antibiotics, hemocoagulase and recombinant human granulocyte colony-stimulating factor. However, the patient did not improve and gradually fell into a coma, with a body temperature of 39°C, a rigid abdominal bulge and a urine volume of <200 ml since the attack. At 13 h post-admission, the serum amylase levels had increased to 938 IU/l, while the serum lipase levels had decreased to 412 IU/l. The urine amylase level was 2,157 IU/l (normal range, <460 IU/l) and the procalcitonin concentration was >200 ng/ml (normal range, <0.5 ng/ml). Ultrasonography revealed large amounts of peritoneum effusion, a poorly visualized pancreas and an empty bladder. Due to these observations, FAP, severe infections and acute renal failure (ARF) were diagnosed. In spite of an attempted rescue of 12 h, the patient succumbed to the disease. Following this, an abdominocentesis was performed and ~300 ml dark red, fetid, chylous ascites was withdrawn. Two days later, a small quantity of gram-negative bacillus growth was identified in an aerobic cultivation of the blood.

## Discussion

In the present study, AP with severe epigastric pain, vomiting and elevated amylase and lipase levels, occurred within two days of completion of the first cycle of chemotherapy. The AP was supervened by shock, hypoxemia and the early stages of ARF, and resulted in mortality. All these symptoms meet the criteria for the diagnosis of FAP. The common etiologies of pancreatitis were eliminated, and a correlation between the incidence of FAP, and pancreatic cancer or chemotherapy, or both was highly suspected. In this case, the interruption of the pancreatic duct observed on MRCP may have been a risk factor for pancreatitis. Previously reported cases of chemotherapy-induced AP have a high variability with respect to the dose administered, onset time following the final dose, whether or not the drug has been used previously and the rechallenge result (Table I). AP is an infrequent complication of chemotherapeutic agents, however, without rechallenge, the diagnosis of chemotherapy-induced pancreatitis is difficult. A number of the reported cases were not rechallenged with the associated chemotherapeutic agents due to being deemed unsafe (5,7,9,10,12,14). While the same chemotherapy was continued with a second episode of AP in the other studies (4,6,8,11,13). For example, in 2010, Yucel and Warmerdam (4) reported the case of a 40-year-old female who developed AP one day following the completion of the third course of capecitibine. The patient recovered five days later owing to the proper treatment. Subsequently, the patient was administered the next course of capecitibine (rechallenge) and again, AP occurred. Therefore, it became clear that capecibine was the cause of AP onset. Only one reported case was rechallenged without the occurrence of AP (3). The patient was a 47-year-old female who developed pancreatitis approximately two months following treatment with capecitabine (2,500 mg/m^2^/day). After healing, the patient was rechallenged with capecitabine at a much lower dose of 1,450 mg/m^2^/day for 14 days. Throughout this course, the results of the laboratory tests remained normal. Therefore, further investigation is required to determine whether or not capecitabine is the cause of AP, as well as if the onset is in a dose-related manner. The chemotherapeutic agents that the current patient received were gemcitabine and capecitabine, however, the drug that caused the episode of AP and how it was caused remain unclear. A total of three cases of capecitabine-induced AP have been previously published (3–5). A study by Chan *et al* (5) proposed that capecitabine-induced hypertriglyceridaemia leading to AP was a possibility. However, in the present patient, all blood lipid levels between admission and mortality were normal. The treatment with capecitabine was discontinued after three days due to the intolerance of the patient to this multidrug therapy. However, the episode occurred two days following the second infusion of gemcitabine. On consideration of the onset time, it is more likely that gemcitabine was the causative agent in this instance of FAP. A literature review by Badalov *et al* (15) revealed that no previous studies have been published with regard to instances of gemcitabine-induced pancreatitis. It is possible that additional cases may be diagnosed if amylase and lipase levels are monitored routinely throughout the duration of gemcitabine-based chemotherapy. With regard to the pathogenesis, if the first episode of AP arises one day following the completion of chemotherapy, it may be due to an allergic reaction or direct toxicity (13). The analysis of mechanism for the incidence of FAP in the present case is as follows: i) Pancreatic cancer with interruption of the pancreatic duct, where the pancreatic juice is not excreted easily; ii) an allergic response or a direct toxic effect due to the chemotherapeutic agents; and iii) digestive disorders due to chemotherapy, nausea and vomiting-induced bile reflux. These, and other unknown possibilities, lead to the activation of trypsin, followed by inflammation of pancreatic tissue caused by their own digestion. Additionally, severe infections secondary to myelosuppression may further aggravate the condition*,* leading to mortality. Clinicians should be aware that life-threatening FAP may occur in patients with pancreatic cancer receiving chemotherapy. Amylase and lipase levels should be checked routinely during or after chemotherapy if abdominal pain, nausea and vomiting appear. Furthermore, once chemotherapy-induced AP has been observed, the patient should not be rechalleged with the chemotherapy.

## Figures and Tables

**Table I:** Main characteristics of the reported cases of chemotherapy-induced AP.

First author/s, year (ref.)	Age/gender	Diagnosis	Drugs/dose, mg/m^2^/days	Prior use	Onset after final dose, days	Highest serum amylase/lipase level, IU/l	Rechallenge	Outcome/duration, days
Jones and Valero, 2003 (3)	47/F	Breast cancer with pleural metastasis	Capecitabine/2,500/14	Yes	60	1,157/115	Negative^a^	Improved/9
Yucel and Warmerdam, 2010 (4)	40/F	Duodenal cancer	Capecitabine/1,250/14	Yes	1	760/NA	Positive	Improved/5
					1^c^	140/NA		Improved/5
Chan *et al*, 2012 (5)	42/F	Colon cancer	Capecitabine/2,000/14	Yes	2	2,046/NA	No attempt	Improved/21
Hoff *et al*, 1997 (6)	74/F	Breast cancer with liver metastasis	Paclitaxel/1,75/3 h	No	1	168/503	Positive	Improved/23
					1^c^	128/586		Deceased/16
Kumar *et al*, 2003 (7)	36/F	Ovarian cancer, stage III	Paclitaxel/1,75/3 h	No	10	1,831/NA	No attempt	Improved/5
Elouni *et al*, 2010 (8)	58/F	Myeloma	Bortezomib/1.3/2^b^	No	2	67/493	Positive	Improved/8
					1^c^	NA/477		Improved/6
Solakoglu *et al*, 2013 (9)	67/M	Multiple myeloma	Bortezomib/NA/NA	No	4	354/523	No attempt	Improved/3
Tester *et al*, 1997 (10)	40/F	Advanced breast cancer	Vinorelbine/NA/NA	Yes	21	109/469	No attempt	Improved/11
Izraeli *et al*, 1994 (11)	16/F	Osteosarcoma with multiple lung, kidney and liver metastases	Ifosfamide/1,800/3 or 1,200/3	Yes	1	1378/554	Positive	Improved/10
					1^c^	1,956/8,340		Improved/10
Gerson *et al*, 1997 (12)	65/F	Small cell lung cancer with right adrenal and brain metastasis	Ifosfamide/1,200/2	No	2	452/402	No attempt	Improved/6
Hung *et al*, 2007 (13)	9/M	Localized osteosarcoma	Ifosfamide/3,000/5 or 2,400/5	Yes	1	1,173/5,027	Positive	Improved/10
					48^c^	879/11,610		Improved/18
Grag *et al*, 2010 (14)	7/F	Wilms tumor, stage 4	Ifosfamide/1,500/3	Yes	1	1,600/NA	No attempt	Improved/3

a The patient was rechallenged at a much lower dose of 1,450 mg/m^2^/day, and did not develop AP.

b With a four day time interval.

c Second occurrence of AP. NA, not available;

AP, acute pancreatitis.
